# Treatment of Glucocorticoid-Induced Osteoporosis and Risk Factors for New Vertebral Fractures in Female Patients with Autoimmune Diseases

**DOI:** 10.1155/2021/5515653

**Published:** 2021-10-25

**Authors:** Koichiro Shinoda, Hirofumi Taki

**Affiliations:** First Department of Internal Medicine, University of Toyama, 2630 Sugitani, Toyama 930-0194, Japan

## Abstract

We aimed to evaluate the compliance of physicians with the 2014 guidelines of the Japanese Society for Bone and Mineral Research, for the prevention and treatment of glucocorticoid (GC) induced osteoporosis (GIO) and to investigate the risk of fracture and other associated risk factors in bisphosphonate-treated patients. We evaluated 90 female patients with nonrheumatoid arthritis autoimmune diseases who received long-term GC treatment (≥12 months). Clinical characteristics, including age, GC dose, history of fragility fractures, osteoporosis treatments, as well as lumbar (L2–L4) and femoral neck bone mineral density, were collected from the patients' medical charts. New vertebral fractures during the study period were evaluated using thoracic and lumbar spine radiographs by quantitative measurements. The GIO score was calculated for each patient according to 2014 Japanese guidelines. Of the 90 patients evaluated, 60 were indicated for osteoporosis treatment, based on the 2014 guidelines of Japan. We observed a high compliance rate, with 93% of patients receiving osteoporosis treatment and 50% receiving bisphosphonates. In total, eight patients developed new vertebral fractures during the study, six of whom received bisphosphonates. In bisphosphonate-treated patients, fracture risk was associated with GC treatment and a lack of active vitamin D3 supplementation. The compliance rate with the updated Japanese 2014 guidelines at our institution was very high. Large randomized controlled trials are needed to confirm our findings that suggest that active vitamin D3 should be used in combination with bisphosphonates for the treatment of GIO to reduce fracture risk.

## 1. Introduction

Glucocorticoids (GCs), which have anti-inflammatory and immunosuppressive effects, are widely used to treat various diseases, including autoimmune disorders. However, long-term GC therapy may have various adverse effects. GC-induced osteoporosis (GIO) is the most common of these, and 30%–50% of patients who receive long-term GC therapy develop fractures [1, 2]. GIO is characterized by a dose-dependent risk of fracture and loss of bone mineral density (BMD) in the lumbar spine and femoral neck that peaks during the first 3–6 months of GC treatment [2]. Fracture risk is higher in patients with GIO than in those with postmenopausal osteoporosis at similar baseline BMD levels, and fractures can occur with minimal or no loss of BMD [3, 4]. Therefore, early preventive measures against GIO are essential in patients under long-term GC therapy.

In 2004, the Japanese Society for Bone and Mineral Research (JSBMR) released clinical guidelines for GIO treatment in patients aged 18 years or older, who planned to use oral GCs for over 3 months [5]. The JSBMR 2004 guidelines recommend antiosteoporosis treatment for patients with previous, or new fragility fractures, as well as those with a BMD ≤80% of the young adult mean (YAM) [5]. However, adherence to these guidelines was only 23.3%, possibly because of the low rate of BMD measurements in clinical settings [6]. In 2014, the JSBMR identified age, GC dose, lumbar spine BMD, and a history of fragility fractures as independent risk factors for new fractures and incorporated these risk factors into a new scoring system to increase compliance [7].

**Figure 1:**
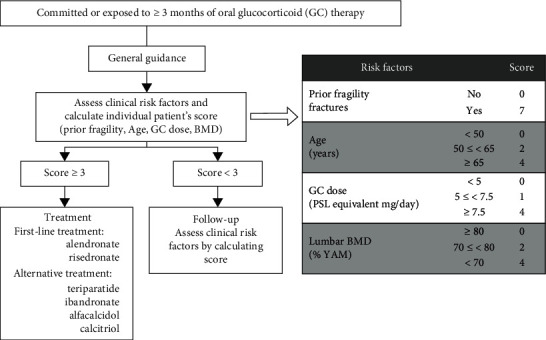
Osteoporosis treatment for patients identified as high-risk for glucocorticoid-induced osteoporosis.

Based on the JSBMR 2014 guidelines, fracture risk is calculated as the sum of the scores for each risk factor, and pharmacological prevention and treatment for GIO are recommended in patients who reach a total score of 3 or more (Figure 1) [7]. Furthermore, patients aged 65 years or older, with prior fragility fractures, receiving a GC dose of 7.5 mg/day or higher, or those with a lumbar spine BMD <70% of YAM, are considered to be at risk for future fractures and should therefore receive treatment for osteoporosis [7]. Treatment with bisphosphonates, such as alendronate, risedronate, and zoledronic acid, with concomitant vitamin D and calcium supplementation, is recommended as the first-line treatment option for GIO [8]. Although all three treatments inhibited lumbar and hip BMD loss, only alendronate and risedronate reduced the rate of vertebral fractures [9–16]. However, people taking bisphosphonates may be more susceptible to osteonecrosis of the jaw (ONJ) [17] and atypical femoral fractures [18, 19]. In addition, although bisphosphonates have not been known to impact pregnancy or cause fetal malformation [20, 21], there is still insufficient evidence for the same, and the American College of Rheumatology does not recommend bisphosphonate treatment for those planning to become pregnant [22]. Alternative treatments for GIO include denosumab [23, 24], teriparatide [25, 26], and active vitamin D3 monotherapy [27]. However, whether clinicians adhere to the JSBMR 2014 guidelines and recommend osteoporosis treatment accordingly remains unknown.

In this observational study, we investigated compliance with the JSBMR 2014 guidelines in patients with autoimmune diseases other than rheumatoid arthritis (RA). In addition, we identified six bisphosphonate-treated patients who developed new vertebral fractures during the study period and determined the fracture risk factors for these patients.

## 2. Materials and Methods

### 2.1. Participants

In this single-center, retrospective study, we examined all female patients with autoimmune diseases other than RA, who visited the author's specialized outpatient clinic at the Department of Rheumatology and Clinical Immunology at Toyama University Hospital, from April to December 2017. A total of 97 patients underwent long-term GC treatment (≥12 months). We included patients who had at least two thoracic and lumbar spine radiographs (frontal and lateral views) and had BMD measurements made by dual-energy X-ray absorptiometry of the lumbar spine and femoral neck, at the initial and final examinations at our hospital. There were no other exclusion criteria. Study approval was obtained from the Ethics Committee of Toyama University Hospital (no. R2017161). The patients provided informed consent via an opt-out form provided on our hospital website.

### 2.2. Data Collection

Data were collected from the patients' medical charts, from the start of GC therapy to December 31, 2017. The data collected included age, underlying disease, disease duration, maximum and current daily dose of GC, osteoporosis treatment and duration, prior fragility fractures, lumbar L2-4 BMD (%YAM), and femoral neck BMD (%YAM). Adverse events due to osteoporosis medication, including ONJ and atypical femoral fractures, were also observed.

New vertebral fractures during the study period were evaluated using thoracic and lumbar spine radiographs (frontal and lateral views), by manual quantitative measurements. The diagnosis of vertebral fractures was based on the criteria for vertebral fractures (1996 version) [28]. For patients who developed new vertebral fractures during the study period, data were collected at the time of fracture, except for BMD, for which data were collected at the final measurement, before the fracture. Data were collected from the time of the final BMD measurement for patients who did not have a new vertebral fracture.

### 2.3. GIO Scoring

For each patient, the GIO score was calculated based on the JSBMR 2014 guidelines [7], using lumbar or femoral neck BMD measurements. New fractures during the study period were not included in the GIO scores.

### 2.4. Statistical Analysis

The clinical characteristics of bisphosphonate-treated patients, with or without fractures were compared using the *t*-test or *χ*^2^-test. Logistic regression analysis was performed using new fractures as the dependent variable and each risk factor as an independent variable. JMP® software (version 13, SAS Institute Inc.) was used for all statistical analyses, and *p* values <0.05 were considered as statistically significant.

## 3. Results

### 3.1. Subject Characteristics

Of the 152 female patients who visited our clinic for autoimmune diseases other than RA between April and December 2017, 90 met the inclusion criteria and were included in our study. The clinical characteristics of the 90 female patients receiving long-term GC treatment (≥12 months) are shown in Table 1. The final GC dose was <5 mg/day in 30%, 5–7.5 mg/day in 40%, and ≥7.5 mg/day in 30% of the patients. Eleven patients had a history of vertebral fracture before the initial examination at our hospital. The mean lumbar and femoral neck BMD measurements recorded at the final observation were 95.7% and 87%, respectively.

### 3.2. JSBMR 2014 Guideline Adherence

To determine the guideline adherence, we first calculated the GIO scores for GC-treated patients using the JSBMR 2014 guidelines (Figure 1) [7]. According to these guidelines, 57 (63%) and 60 (66%) patients had a GIO score of 3 or higher when scores were based on lumbar or femoral neck BMD measurements, respectively, and were therefore indicated for osteoporosis treatments (Table 2).

Of the 60 patients with a GIO score of 3 or higher, 56 (93%) received osteoporosis treatment (Table 2). Thirty patients received bisphosphonate treatment, with 15 of these patients receiving concomitant active vitamin D3 (alfacalcidol or eldecalcitol), the only approved vitamin D3 supplement in Japan. In addition, 12 patients received denosumab, 3 received daily subcutaneous injections of teriparatide, and 11 received active vitamin D3 (alfacalcidol or eldecalcitol) monotherapy. Only four patients (6%) did not receive any osteoporosis treatment.

Next, we investigated the reasons behind why some patients did not receive osteoporosis treatment and why some received only active vitamin D3. In the untreated patients, two refused treatment, whereas in the other two cases, the physician did not prescribe osteoporosis medication, for which no reason was provided. Of the 11 patients who received only active vitamin D3, two were planning to become pregnant, three were receiving dental treatments, two had experienced side effects when using bisphosphonates, and the remaining four were not prescribed bisphosphonates by the attending physician.

Adverse events for patients who received osteoporosis medication included two patients with ONJ (one receiving bisphosphonates and one receiving denosumab) and one patient with an atypical femoral fracture (receiving bisphosphonates). The patients with ONJ had been on glucocorticoid therapy for 25 and 19 years and were treated with 15 mg or 12.5 mg/day of prednisolone, respectively, when they developed ONJ. The patient with an atypical femoral fracture had been on glucocorticoid therapy for 11 years and was treated with 5 mg/day of prednisolone, after which she developed an atypical femoral fracture.

### 3.3. Development of New Vertebral Fractures

An additional eight patients developed vertebral fractures during the study period (Table 3), and no other fractures were detected in this patient population. Of these, two patients received a high dose of GC (≥10 mg/day), but had not been treated with bisphosphonates. A 34-year-old patient avoided bisphosphonate treatment because of pregnancy concerns, whereas a 49-year-old patient did not receive bisphosphonate treatment at the physician's discretion. The remaining six patients developed new vertebral fractures after bisphosphonate treatment. Vertebral fractures occurred in high-risk patients with a high GIO score of ≥4 points.

### 3.4. Vertebral Fracture Risk Factors in Bisphosphonate-Treated Patients

To determine the vertebral fracture risk factors in bisphosphonate-treated patients, we compared the clinical characteristics of the six bisphosphonate-treated patients with new vertebral fractures with the 24 bisphosphonate-treated patients without new vertebral fractures (Table 4). There was a trend towards an increase in age and disease duration in patients with vertebral fractures compared to those without vertebral fractures; however, this difference was not statistically significant. Although there were no differences in the maximum GC dose, the current GC dose was significantly higher in the vertebral fracture group than in the nonvertebral fracture group. In addition, a significantly higher proportion of patients in the nonvertebral fracture group were prescribed concomitant active vitamin D3 than those in the vertebral fracture group. Although the lumbar and femoral neck BMD measurements did not differ between the two groups, the GIO scores were significantly higher in the vertebral fracture group than in the nonvertebral fracture group.

Multiple logistic regression analysis identified current GC dose, concomitant active vitamin D3 use, and GIO scores, as factors predicting future vertebral fractures (Table 4). Interestingly, the risk of developing vertebral fractures was 19 times higher in patients receiving bisphosphonate monotherapy than in those receiving bisphosphonates with concomitant active vitamin D3. Unfortunately, due to the small sample size, multiple logistic regression analysis could not be performed for prior fragility fractures.

## 4. Discussion

GCs are commonly used to treat many autoimmune diseases. Although, long-term GC use is associated with various side effects, we found that 97 out of 152 patients (63.8%) with autoimmune diseases, excluding RA, continued to receive GC treatment in our clinic. The side effects of GC are diverse and can affect the whole body; among them, GIO and secondary vertebral fractures are the most severe adverse events that significantly affect a patient's quality of life [2]. Therefore, in this study, we investigated the treatment of osteoporosis in GC-treated patients with autoimmune diseases. We omitted RA from our analysis because bone destruction and systemic osteoporosis are characteristic of RA [29] and could, therefore, confound our analyses.

Kirigaya et al. [6] investigated the compliance rate to the JSBMR 2004 guidelines based on the health insurance claims database and found that only 23.3% of the 2,368 patients indicated for osteoporosis treatments were prescribed the recommended medication. Based on this information, the JSBMR updated its guidelines in 2014 to include multiple independent risk factors for GIO to make the guidelines more adaptable in the clinical setting [7]. Our study is the first to evaluate compliance with the JSBMR 2014 guidelines. We found that 63%–66% of patients in our study scored 3 or higher and were indicated for osteoporosis treatment. Of these patients, 93% received osteoporosis treatments and 50% received bisphosphonate treatment, demonstrating a much higher compliance rate compared to the JSBMR 2004 guidelines [6]. Interestingly, only two patients did not receive osteoporosis treatment for unknown reasons. This suggests that the updated JSBMR 2014 guidelines are more easily incorporated into clinical practice than the JSBMR 2004 guidelines. However, the compliance rates for the JSBMR 2004 guidelines differed across various departments, with departments of surgery and otolaryngology exhibiting lower adherence compared to the department of internal medicine [6]. Our report is based on the analysis of a single department in a single medical institution. Further studies are needed to investigate the JSBMR 2014 guideline compliance rates across all the clinical departments.

Although there have been reports of ONJ in patients with osteoporosis receiving antiresorptive treatments, the incidence is low, with rates below 0.001% for bisphosphonate-treated patients [17] and 0.08% for denosumab-treated patients [30]. Surprisingly, in our study, one (3.3%) patient taking bisphosphonate and one (8.3%) patient taking denosumab developed ONJ, which is a relatively high incidence rate compared to other studies of antiresorptive-treated patients. Some studies found that GC use was a risk factor for ONJ [31, 32], possibly because of its immunosuppressant effects, which include slow wound healing and changes in the oral microflora, thereby increasing the risk of infection. However, GC use was not associated with ONJ in patients with RA [33]. Although we observed a small sample size of patients, our data suggest that it is necessary to analyze the risk of ONJ during GC use in non-RA autoimmune diseases.

During the study period, eight patients developed new vertebral fractures, six of whom received bisphosphonates. Compared to the bisphosphonate-treated patients who did not develop new vertebral fractures, those who developed vertebral fractures had a significantly higher current GC dose, which is consistent with the findings of other studies [34]. This suggests that patients taking a higher dose of GC are at a higher risk of vertebral fractures; however, we did not measure the cumulative GC dose, which is also associated with an increased fracture risk [35].

In addition, a significantly lower proportion of patients with new vertebral fractures received concomitant active vitamin D3 compared to those without new vertebral fractures. Importantly, the fracture risk for patients taking bisphosphonates was 19 times higher than for those who did not receive active vitamin D3. These results are consistent with findings from a three-arm randomized controlled study (EDITOR-J Study), which found that the combined use of alfacalcidol and alendronate significantly increased lumbar BMD and reduced the incidence of fractures in patients with GIO compared to treatment with alfacalcidol or alendronate alone [36]. The mechanism for the efficacy of the combined use of alfacalcidol and alendronate is thought to be that active vitamin D3 ameliorates secondary hyperparathyroidism caused by glucocorticoid-induced inhibition of intestinal Ca absorption, inhibition of renal tubular calcium reabsorption, and shortage of Ca mobilization following alendronate monotherapy. In addition, the effectiveness of bisphosphonates was positively correlated with serum vitamin D levels [37]. As vitamin D insufficiency is common in postmenopausal women [38], vitamin D supplementation is recommended for overall bone health [39]. Guidelines and recommendations for the prevention and treatment of GIO, developed by the American College of Rheumatology, suggest that all patients taking glucocorticoids maintain a vitamin D intake of 600 to 800 international units/day through either diet and/or supplements [22]. Guidelines on the prevention and treatment of GIO by the French National Authority for Health recommend that loading and maintenance doses of vitamin D should be administered to elevate the serum 25-OH vitamin D level above the target of 30 ng/mL in patients with vitamin D insufficiency or deficiency [40]. In Japan, the JSBMR 2014 guidelines also recommend improving nutrition, including dietary calcium and vitamin D intake, maintaining healthy body weight, ceasing smoking and alcohol intake, and exercising, for the management of GIO, which is similar to that recommended for the management of primary osteoporosis. However, there are no approved native vitamin D agents (ergocalciferol and cholecalciferol) listed for use with bisphosphonates, and there is no custom of taking native vitamin D supplements; therefore, approved active vitamin D3 agents (alfacalcidol or eldecalcitol) have been used in combination with bisphosphonates. Together, these data support the use of active vitamin D3 in combination with bisphosphonates for the treatment of GIO, especially in patients with a high fracture risk. However, in our study, only 50% of bisphosphonate-treated patients also received active vitamin D3.

The mean BMDs in the vertebral fracture group were 84.3% and 83.2% in the lumbar and femoral neck, respectively, indicating a relatively normal bone mass. Furthermore, there was no significant difference in BMD between the vertebral and nonvertebral fracture groups. These findings are consistent with studies demonstrating that fractures can occur with minimal or no loss of BMD in patients with GIO [3, 4]. Our study provides further evidence that BMD should not be the only indicator of osteoporosis treatment in patients with GIO. In addition, the GIO scores calculated on the basis of the JSBMR 2014 guidelines were significantly higher in the vertebral fracture group than in the nonvertebral fracture group, demonstrating the effectiveness of the scoring system in predicting fracture risk.

This study has some limitations. First, as our study was a single-center analysis, we could not determine whether the compliance rate of the JSBMR 2014 guidelines was superior to that of the JSBMR 2004 guidelines. Second, our sample size was small, which may have resulted in selection bias, and large-scale prospective studies encompassing multiple clinics are needed to confirm our findings. Third, we did not measure serum vitamin D levels in these patients. Finally, treatment agents and autoimmune diseases varied considerably. However, previous studies have demonstrated similar risks of GIO and fractures, in many autoimmune diseases [7].

## 5. Conclusions

This study demonstrated a high compliance rate with Japan's GIO treatment guidelines. Based on our results, it might be recommendable that active vitamin D_3_ be used to reduce the fracture risk associated with bisphosphonates to treat GIO. Large randomized controlled trials should be carried out to confirm our data.

## Figures and Tables

**Table 1:** Participant characteristics.

	Total (*n* = 90)
Age (years), mean (SD)	52.9 ± 14.5
Sex, female (%)	90 (100)

Underlying diseases, no. (%)	
SLE	36 (40.0)
Inflammatory myopathy	14 (15.5)
Scleroderma	9 (10)
MCTD	8 (8.9)
Sjogren's syndrome	5 (5.6)
Vasculitis	6 (6.7)
Others	12 (13.3)

Current glucocorticoid dose, no. (%)	
<5 mg/day	27 (30)
5 ≤ *x* < 7.5 mg/day	36 (40)
≥7.5 mg/day	27 (30)
Prior fragility fracture (s), no. (%)	11 (12.2)

BMD (% YAM), mean (SD)	
Lumbar spine	95.7 (12.7)
Femoral neck	87 (12)

Glucocorticoid-induced osteoporosis score, lumbar BMD	
<3, no. (%)	33 (37)
3–5, no. (%)	25 (28)
≥6, no. (%)	32 (36)

Glucocorticoid-induced osteoporosis score, femoral neck BMD	
<3, no. (%)	30 (34)
3–5, no. (%)	23 (26)
≥6, no. (%)	37 (40)

SLE, systemic lupus erythematosus; MCTD, mixed connective tissue disease; YAM, young adult mean. Glucocorticoid-induced osteoporosis scores were determined as described previously [7].

**Table 2:** Osteoporosis treatment for patients identified as high-risk for glucocorticoid-induced osteoporosis.

	Prescribed no. (%)
Osteoporosis treatment	56 (93)
Bisphosphonates	30 (50)
With active vitamin D_3_	15 (25)
Without active vitamin D_3_	15 (25)
Teriparatide	3 (5)
Denosumab with vitamin D_3_	12 (20)
Active vitamin D_3_ monotherapy	11 (18)
Alfacalcidol	7 (12)
Eldecalcitol	4 (6)
Untreated	4 (6)

**Table 3:** Characteristics of patients who developed new vertebral fractures during the study period.

Patient	1	2	3	4	5	6	7	8
Age at fracture, (years)	34	49	73	68	75	52	65	44
Underlying disease	SLE	MCTD, HPS	PM	ILD	PMR	SLE, APS	SLE	TAK
Age at onset (years)	13	40	72	64	72	23	35	22
Disease duration (years)	21	9	1	4	3	29	30	22
Max. glucocorticoid dose (mg)	30	90	65	30	15	40	15	40
Glucocorticoid dose at fracture (mg)	20	10	13	6	5	8	10	13
Osteoporosis treatment	None	D_3_	BisP	BisP	BisP	BisP + D_3_	BisP	BisP
Osteoporosis treatment duration (months)	NA	6	10	19	26	114	42	180

BMD, % YAM								
Lumbar spine	65	93	84	73	86	91	73	99
Femoral neck	60	84	83	75	81	87	90	83

Glucocorticoid-induced osteoporosis score								
Lumbar spine	8	4	8	7	5	6	10	11
Femoral neck	8	4	8	7	5	6	8	11
Prior fragility fracture	No	No	No	No	No	No	No	Yes

SLE, systemic lupus erythematosus; MCTD, mixed connective tissue disease; HPS, hemophagocytic syndrome; PM, polymyositis; ILD, interstitial lung disease; PMR, polymyalgia rheumatica; APS, antiphospholipid syndrome; TAK, Takayasu arteritis; BMD, bone mineral density; YAM, young adult mean; D_3_, active vitamin D_3_; BisP, bisphosphonate.

**Table 4:** Comparison of patient characteristics in patients who received bisphosphonate treatments with and without new vertebral fractures.

	Without fracture (*n* = 24)	With fracture (*n* = 6)	*p* value (*t*-test)	Unadjusted OR (95% CI)	*p* value (logistic regression)
Age, (years)	56 (13.1)	63 (12.4)	0.25	1.05 (0.97-1.14)	0.215
Disease duration (years), mean (SE)	9.5 (7.8)	15 (13.4)	0.19	1.07 (0.97-1.17)	0.189
Max. glucocorticoid dose (mg)	33.1 (17.7)	34 (18.8)	0.90	1.003 (0.95-1.06)	0.895
Current glucocorticoid dose (mg)	6.3 (2.7)	9.3 (3.2)	0.02	1.45 (1.02-2.07)	0.022
Combined vitamin D_3_, no. (%)	19 (79.2)	1 (16.7)	0.0037	19 (1.79-201.68)	0.015
Prior fragility fracture, no. (%)	0 (0)	1 (16.7)	0.0419	NA	NA

BMD, % YAM					
Lumbar spine	93.0 (2.4)	84.3 (5.6)	0.10	0.93 (0.85-1.012)	0.076
Femoral neck	85.6 (13.3)	83.2 (5.2)	0.67	0.98 (0.91-1.06)	0.66

Glucocorticoid-induced osteoporosis score					
Lumbar spine	4.3 (1.8)	7.8 (2.3)	0.0006	2.15 (1.18-3.92)	0.001
Femoral neck	5.0 (2.2)	7.5 (2.1)	0.0213	1.54 (1.01-2.34)	0.027

Data are presented as mean (SD) unless otherwise stated. BMD, bone mineral density; D_3_, active vitamin D_3_; YAM, young adult mean.

## Data Availability

The datasets associated with the current study are available from the corresponding author upon reasonable request.
